# Human toxoplasmosis in Mozambique: gaps in knowledge and research opportunities

**DOI:** 10.1186/s13071-020-04441-3

**Published:** 2020-11-11

**Authors:** Leonardo Manuel, Gabriela Santos-Gomes, Emilia V. Noormahomed

**Affiliations:** 1 Faculty of Health Sciences, Universidade Lurio, Nampula, Mozambique; 2 Global Health and Tropical Medicine (GHTM), Instituto de Higiene e Medicina Tropical (IHMT), Universidade Nova de Lisboa (UNL), Lisbon, Portugal; 3 Department of Microbiology, Faculty of Medicine, Universidade Eduardo Mondlane (UEM), Maputo, Mozambique; 4 Department of Medicine, Infectious Disease Division, University of California, San Diego, USA; 5 Mozambique Institute for Health Education and Research (MIHER), Maputo, Mozambique

**Keywords:** *Toxoplasma gondii* infection, HIV-infected patients, Congenital toxoplasmosis, Ocular toxoplasmosis, Mental disorders, South east African countries, Mozambique

## Abstract

Toxoplasmosis is a parasitic zoonotic disease caused by *Toxoplasma gondii* that afflicts humans worldwide and wild and domestic warm-blooded animals. In immunocompetent individuals, the acute phase of infection presents transient low or mild symptoms that remain unnoticed. In immunocompromised patients, *T. gondii* is a life-threatening opportunistic infection, which can result from the reactivation of latent infection or primary infection. Moreover, congenital toxoplasmosis, which results from the transplacental passage of tachyzoites into the fetus during a pregnant primary infection, can lead to miscarriage, stillbirth, or ocular and neurologic disease, and neurocognitive deficits in the newborns. Thus, the present review aims to address the current knowledge of *T. gondii* infection and toxoplasmosis in Africa and especially in Mozambique, stressing the importance of identifying risk factors and promote awareness among the health care providers and population, assessing the gaps in knowledge and define research priorities. In Mozambique, and in general in southern African countries, clinical disease and epidemiological data have not yet been entirely addressed in addition to the implications of *T. gondii* infection in immunocompetent individuals, in pregnant women, and its relation with neuropsychiatric disorders. The main gaps in knowledge in Mozambique include lack of awareness of the disease, lack of diagnostic methods in health facilities, lack of genetic data, and lack of control strategies.
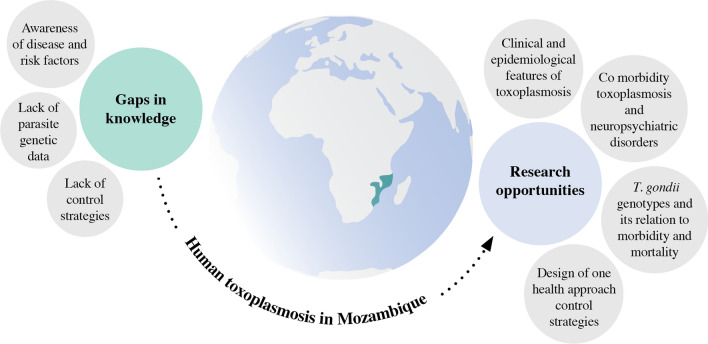

## Background

Toxoplasmosis is a zoonotic disease caused by the parasite *Toxoplasma gondii*, a cosmopolitan intracellular protozoan. This parasite can infect a wide range of warm-blooded animals, including humans who act as intermediate hosts, supporting the asexual phase of the *T. gondii* life-cycle. Cats and wild felines have been considered definitive hosts since the sexual reproductive phase of the *T. gondii* life-cycle is restricted to these animals. People may become infected through the ingestion of raw or undercooked meat containing cyst, or by food and water contaminated with highly resistant and easily dispersed *T. gondii* oocysts from feline feces [1–3]. It seems that one to ten sporulated oocyst is enough to cause infection, giving rise to the asexual phase of the *T. gondii* life-cycle [4, 5]. Infection also can be acquired by cysts after organ transplantation and by tachyzoites, which can cross the placenta during pregnancy, causing congenital toxoplasmosis and through blood transfusion [1, 2, 6]. Globally, it is anticipated that one-third of the world population is infected with *T. gondii* and that the prevalence of infection varies between 10–80%, depending on local culture, eating habits, and climate [6–8]. In South America and tropical Africa, the prevalence of the disease is very high, with more than 50% of people infected, while in Europe, North America, and Southeast Asia the prevalence rates range from 7% to 50% [3, 9, 10]. Studies conducted in several countries of Southeast Africa, such as Zambia, South Africa, Eswatini (former Swaziland), Zimbabwe, Angola, Namibia, Tanzania, Madagascar, Uganda, Kenya, Ethiopia, and Mozambique, indicate prevalence of *T. gondii* infection that ranges from 4% to 93% in the general population [11–13]. Signs, symptoms, and the severity of *T*. *gondii* infection differs according to the immune status of the individual, the age in which the infection was acquired, and the genotype of the parasite involved [2, 3, 14–16]. In immunosuppressed patients due to human immunodeficiency virus (HIV) or immunosuppressive therapy, toxoplasmosis is considered a life-threatening parasitic disease. Despite the growing numbers of drug-immunosuppressed patients and the few available studies, these patients can also be at risk of developing toxoplasmosis, in particular, the transplanted patients [17, 18]. *Toxoplasma gondii* genotyping studies recognize three major subtypes identified as subtype I, subtype II, and subtype III. Altogether they account for 95% of isolates from North America and Europe, each leading to differences in disease severity [2, 3]. In these regions, the majority of cases of congenital toxoplasmosis and toxoplasmosis infection in HIV immunosuppressed individuals are mainly caused by type II strains. However, most of the isolates from South America, Africa, and Asia do not fit into the three major lineages, except type III, which is really cosmopolitan and commonly found in animals [2, 19]. Atypical, exotic, recombinant, or non-archetypal genotypes were found in other continents and the characterization of the strains by multilocus polymerase chain reaction-restriction fragment length polymorphism (PCR-RFLP), using ten genetic markers revealed 18 different genotypes. Together they account for 5% of infections, generating more virulent parasites due to its genetic diversity and the consequent increase of disease severity [3, 14, 20]. A large meta-analysis and a prospective cohort study showed a higher risk of ocular *Toxoplasmosis* in children from Brazil and Colombia than in European children (47% *versus* 14%). Furthermore, ocular lesions were large, numerous, and more likely to affect the retina that according to several authors may be explained by the predominance of atypical *T. gondii* strains in Latin America [21–23]. There are multiple tools available for the diagnosis of *T. gondii* infection, particularly serological, molecular, and imaging techniques. Serological assays allow the detection of *T. gondii* specific antibodies, immunoglobulin M (IgM) and immunoglobulin G (IgG). Usually, these tests possess high sensitivity and specificity due to antigen standardization and good assay reproducibility in immunocompetent individuals [24]. Molecular biological tests based on polymerase chain reaction (PCR) allow detection of parasite deoxyribonucleic acid (DNA) and identification of genetic variants, while imaging techniques, such as computed tomography and magnetic resonance imaging, that have been employed to evaluate expansive brain lesions of cerebral toxoplasmosis, present high sensitivity, but low specificity and should be used in combination with other diagnostic methodologies [2, 3, 25–27]. More recently, the use of recombinant antigens has been proposed as an alternative to conventional serological assays because of its limited value, especially in immunosuppressed patients [3, 28, 29]. Patients with confirmed toxoplasmosis have multiple treatment options, depending on immune competence and disease severity. However, most of the drugs used to treat toxoplasmosis are effective against tachyzoites, the acute morphological form of *T. gondii*, but do not seem to eradicate the encysted bradyzoites forms (chronic phase). Pyrimethamine combined with sulfadiazine and trimethoprim-sulfamethoxazole combined with spiramycin are the main choice for the clinical treatment of toxoplasmosis [30]. To prevent parasite transmission from a pregnant woman to her fetus spiramycin is the drug of choice. For congenital toxoplasmosis, pyrimethamine and sulfadoxine together with folinic acid to prevent bone marrow suppression are the recommended drugs to treat the newborn, while a combination of pyrimethamine, azithromycin, and corticosteroids is recommended for treating ocular toxoplasmosis [27]. Despite the limited reports available on the relative importance of toxoplasmosis in Africa and particularly in Mozambique, where the studies on human *T. gondii* infection are scarce, this review aims to: (i) summarize and critically examine the most relevant aspects of *Toxoplasma* infection and toxoplasmosis in Africa and, specifically in Mozambique; (ii) identify gaps of knowledge; (iii) highlight the research opportunities and reflect on its implications for the population well-being and for the socio-economic development of Mozambique.

## Toxoplasmosis in immunosuppressed patients

In HIV-immunosuppressed patients and individuals undergoing cancer treatment or organ transplantation, *Toxoplasma* infection can become a severe opportunistic disease, which can result from a primary infection, but most of the time results from reactivation of an earlier acquired infection. Reactivation of bradyzoites (the dormant forms of this parasite) and differentiation in tachyzoites occurs as a consequence of patient's reduced immunocompetence, leading to tissue injury [31, 32]. In sub-Saharan Africa, where the majority (70%) of HIV-infected people live [33], patients are commonly diagnosed with HIV after developing cerebral toxoplasmosis. It is estimate that this parasitic disease is indicative of HIV infection in 35% of patients and an acquired immunodeficiency syndrome (AIDS) defining event in 75% of the cases [3, 32, 34, 35]. With access to combination antiretroviral therapy (cART), the incidence of encephalitis caused by *T. gondii* in HIV^+^ patients have reduced dramatically. Before the anti-retroviral treatment era, rates of cerebral toxoplasmosis in the USA and the UK ranged between 16 and 40%, in Brazil 50 to 80%, in France 75 to 90%, and in Spain, it was about 60% [36]. In Ethiopia, a serological study in HIV-infected patients found *Toxoplasma* infections ranging from 3% to 97% [37, 38]. In Uganda anti-*T. gondii* antibodies were detected in 54% of HIV^+^ patients and 23% present parasites in the peripheral blood [39], pointing for an acute infection that possibly represents a reactivation. On the contrary, in Johannesburg (South Africa), the prevalence of latent *Toxoplasma* infection was lower in HIV infected patients, ranging between 8% and 18% [40, 41]. Moreover, in Nigeria, 85.5% of HIV/AIDS patients that were under cART present latent *Toxoplasma* infection (seropositive for *T. gondii* IgG) and almost 40% (39.7%) exhibited focal neurological signs [42]. Taken together, these findings indicate that in the African continent, there is a high variability *T. gondii* infection prevalence, which can go from residual values to very high levels. These differences can be associated with the socioeconomic conditions, including the efficacy of health systems and health education and population traditional culture, values, customs, and beliefs.

Although several studies argued for the role of genotypes in the clinical expression of human toxoplasmosis and the geographical structure of *Toxoplasma* across continents, genetic data concerning *T. gondii* isolates from Africa are scarce. In a study performed in HIV^+^ patients from Uganda, the genotype II (12/22) was the most commonly found, followed by the type I genotype (5/22). The less representative genotypes were non-III (3/22) and type III (2/22) [39].

## Toxoplasmosis in neonates and infants

Women at risk of transmitting congenital toxoplasmosis include immunocompetent women when becoming newly infected during pregnancy or challenged with atypical parasite strains, as well as, immunosuppressed mothers with HIV/AIDS when the reactivation of bradyzoites occur during pregnancy [2, 15, 43]. Women that have acquired *T. gondii* infection before pregnancy have a limited risk of inducing a congenital infection. Primary infection during pregnancy does not cause specific symptoms, being unnoticed in most of the women, or lead to some transient low to mild symptoms that usually are not taken into consideration by the patient. In African countries, dating *Toxoplasma* infection during pregnancy is difficult, and the use of specific serology and the respective follow-up is often not extended to all pregnant women [44].

According to earlier studies in southern Africa, *T. gondii* seroprevalence among pregnant women ranges between 15 and 23% [39, 45, 46] and HIV-*Toxoplasma* co-infection was about 8% [47]. However, in sub-Saharan Africa, more recent studies among pregnant women pointing through *T. gondii*-seroprevalences ranging between 5.9% and 85.5%. [48, 49]. As a consequence of the very high levels of *T. gondii* transmission among the Nigerian population (78%), *Toxoplasma* infection during pregnancy was about 30% [50, 51], representing a serious threat for congenital infection.

The risk factors for becoming infected have been identified. Eating raw meat, unwashed fresh vegetables or fruits, and undercooked food, drinking unpasteurized milk, and the proximity to cats seems to be predictors of possible infections [44]. Even so, the prevalence of *Toxoplasma* infection in pregnancy appears to be an underestimated public health concern in Africa, highlighting the urgent need for further research. Furthermore, the awareness of toxoplasmosis and its mode of transmission among women and, in particular, pregnant women seems to be limited [12, 52].

The risk of vertical transmission to the fetus, as a consequence of crossing the placental barrier by tachyzoites, increases during pregnancy, and about 60% to 81% of the infections by *T. gondii* occur during the last trimester [2, 53]. However, the disease is more severe in the early stages of pregnancy, depending on parasite virulence and of the infected *T. gondii* genotype. In the first trimester, the rate of infection can range from 15% to 25% [10, 25, 54–57]. Congenital toxoplasmosis has been associated with a wide range of adverse outcomes, which includes spontaneous miscarriage, stillbirth, ocular disease, and neurologic and neurocognitive deficits. The most frequent presentation of congenital toxoplasmosis comprises of hydrocephalus, chorioretinitis, and cerebral calcifications. In up to 80% of cases, the infection remains asymptomatic after birth, but infants may later present mental retardation and learning and visual disabilities [2, 12, 53]. In upper-middle-income countries, the incidence of congenital hydrocephalus is at 0.5 cases per 1000 live births, whereas in Africa and Latin America the incidence of neonatal hydrocephalus was estimated to be around 145 and 316 per 100.000 births, respectively [58, 59]. In Nigeria it was determined that abortion occurred in 41.6% to 60% of pregnant women presenting anti-*Toxoplasma* antibodies, stillbirth happened in 6.8% to 61.5% of the cases, and neonatal death in 62.5%. Ocular problems occurred in 29.4% of newborns [60–62]. Torgerson and Mastroiacovo [63] estimated per 1000 live births the incidence of congenital cases accounting for 2–2.4 to 13–15 of all Disability-Adjusted Life Years (DALYs) for the African continent, despite several African countries have not reported cases of congenital toxoplasmosis or seroprevalence data. Infants can also acquire primary *Toxoplasma* infection after birth and develop severe disease [3, 35, 64].

In a recent study in Tunisia, *T. gondii* isolated from the amniotic fluid and placenta of women that had an acute infection during pregnancy was revealed to be of the type II genotype [65]*.* However, no other published studies on *T. gondii* genotypes occurring on the African continent were found, especially in sub-Saharan African countries.

## *Toxoplasma* infection in immunocompetent patients

Studies conducted in immunocompetent individuals living in European countries or North America found that primarily acquired *T. gondii* infection is asymptomatic and self-limited in more than 80% of individuals [3, 66]. In a few cases, patients may present fever or cervical lymphadenopathy, sometimes associated with myalgia, asthenia, or other non-specific clinical signs that can be misdiagnosed with different clinical conditions, exhibiting similar symptoms. The knowledge of the neuropathology caused by *Toxoplasma* is progressing. [3, 57, 67–69]. The immune response of chronically infected patients is mainly characterized by interferon-γ production which induces the activation of indoleamine-2,3-dioxygenase leading to tryptophan (amino acid essential for serotonin biosynthesis) depletion, increase of kynurenic acid, and decrease of serotonin. Moreover, high tyrosine hydrolase activity directs dopamine release. Thus, high levels of kynurenic acid and dopamine, along with low amounts of serotonin are associated with cognitive dysfunctions [3].

Schizophrenia is a serious psychiatric disorder with a lifetime prevalence of approximately 1% and is rated as the 9th most common cause of disability all over the world [67]. Since 1953, more than 19 studies were done in patients with schizophrenia and other severe psychiatric disorders, testing for antibodies against *T. gondii*. Of these, 18 studies found patients with a higher frequency of anti-*T. gondii* antibodies, and in 11 out of 18, the association was statistically significant [57]. These conditions are indicated as the leading cause of disability in the world, accounting for 22.7% of DALYs [69].

A systematic review on the relationship between *Toxoplasma* infection and epilepsy concluded that this parasitic infection should be an epilepsy risk factor and that there is a need to conduct more studies to determine the real impact of *T. gondii* infection on epilepsy [70, 71]. Moreover, studies conducted in Turkey and USA, in patients with cryptogenic epilepsy which aimed to evaluate its possible relationship with *T*. *gondii* found 54% and 75% of these patients infected with *Toxoplasma*, respectively [72, 73].

In Southeast Africa, there is a scarcity of studies aiming to define the relationship between epilepsy and *Toxoplasma* infection. A multicenter study conducted in Kenya, South Africa, Uganda, Tanzania, and Ghana in a total of 1711 individuals with active convulsive epilepsy found an odds ratio of 1.39 with previous exposure to *T. gondii* [74]. Moreover, studies from Kenya and other African countries concluded that in addition to *Toxoplasma* infection, malaria, onchocerciasis, neurocysticercosis, and toxocariasis also might be involved in the pathogenesis of epilepsy [75, 76]. There is also evidence of possible association with some neurodegenerative disorders, such as Parkinson and Alzheimer diseases, in which the prevalence of *Toxoplasma* infection is around 85% and 66%, respectively [3, 77–81]. Suicidal behaviour is also a common problem in southeast Africa. A study from South Africa found that 3.2% of adolescents attempted to commit suicide, 5.8% planned, and 7.2 reported ideation [82]. In Zambia, acute psychotic syndrome is the most common outpatient diagnosis, followed by schizophrenia, substance use disorder, and dementia [83]. Although *T. gondii* infection was considered a potential risk factor for suicide attempts [84], no studies exist reporting possible links between suicide attempts and *Toxoplasma* infection in Africa.

## Burden of pathological disorders related possibly to *Toxoplasma* infection in Mozambique

Mozambique is a low-income country located in Southeast Africa, with 28.8 million people, a child mortality rate of 57.9‰, an adult literacy rate of 60.7%, and with most of the population (about 66%) living in rural areas [85]. According to the United Nations Development Programme [86], this country ranks 180th on the human development index, which points towards a high unmeet in health care performance, low education levels, and basic living standards [87]. The national health system covers only 50% of the population, and according to the Mozambique Poverty Reduction Action Plan 2011–2014 [88] 65% of the population have access to a health unit facility within 45 min walking distance of their homes. Despite natural fluctuations according to geographical settings, tuberculosis, HIV, malaria, neglected tropical diseases, in addition to respiratory and diarrheal diseases, still are significant causes of morbidity and mortality in Mozambique and among Southeastern African countries [87, 89–91] Furthermore, non-infectious diseases, including cardiovascular diseases, cancer, chronic respiratory diseases, and diabetes also accounts for disease burden in Mozambique [92].

In 2018, the prevalence of HIV in Mozambique was 12.6% in the age group of 15 to 49 years-old and 45,000 AIDS deaths [93], with nearly 2.2 million people living with HIV infection [94, 95]. Together with some of the neighboring countries, such as Eswatini, South Africa, Zimbabwe, Zambia, Malawi, and Tanzania, Mozambique is among the top ten countries presenting the highest prevalence of HIV in the world, which ranges from 6.5% to 27.2% [91].

*Toxoplasma gondii* infection can also be related to mental disorders in immunocompetent individuals and in immunocompromised patients, in addition to the neurologic disorders of congenital toxoplasmosis. There was a significant improvement in the Mozambique mental health services, reaching rates of 0.59 mental health outpatient facilities and 0.09 psychiatric hospitals for 100,000 persons [96]. Mozambique has a suicide rate of 4.9 and a total of 1412 suicides per year, being in the 134 position in the global suicide rank [97]. However, this country has no published national suicide statistics. Even so, a cross-sectional study performed in unnatural death, recorded from 2000–2009 at the Forensic Services in Maputo Central Hospital, reported 9% of suicide [98]. Epilepsy affects 50 million people worldwide, and about 80% live in low-income countries [99]. According to a previous study, Mozambique has a prevalence of 1.6% for epilepsy, pointing out to more than 400,000 individuals living with epilepsy [100]. However, most of these patients seemed to be children and adolescents [101]. Hydrocephalus is a serious problem in sub-Saharan Africa and despite there is little information about this disorder, it was predicted that in Mozambique an incidence of 2900 to 4800 cases of neonatal hydrocephalus occur per year [102]. Despite the high rates of HIV infection in Southeast Africa, including Mozambique, the high incidence of hydrocephalus in sub-Saharan Africa and Mozambique, and the high prevalence of mental disorders, *Toxoplasma* infection still is overlooked in Mozambique. According to the International Agency for the Prevention of Blindness [103] in Mozambique, sight disease affects more than 300,000 persons from Maputo (capital of Mozambique) and Inhambane province, most of the cases are reported as cataract, glaucoma, and trachoma. Despite these numbers, there is no study or report about the incidence of ocular toxoplasmosis in Mozambique.

Though, little is known about the burden of toxoplasmosis in Southeastern Africa and Mozambique. In particular, the identification of risk factors, the genetic diversity of the parasite, as well as, *Toxoplasma* association with HIV, was not examined, the incidence of *Toxoplasma* infection in pregnant women, and the relationship with hydrocephalus and neuropsychiatric disorders, such as epilepsy, schizophrenia, suicidal behaviors, mood disorders, obsessive-compulsive disorder, and generalized anxiety [104] among other conditions, was not examined.

## Toxoplasma infection in Mozambique

**Fig. 1:**
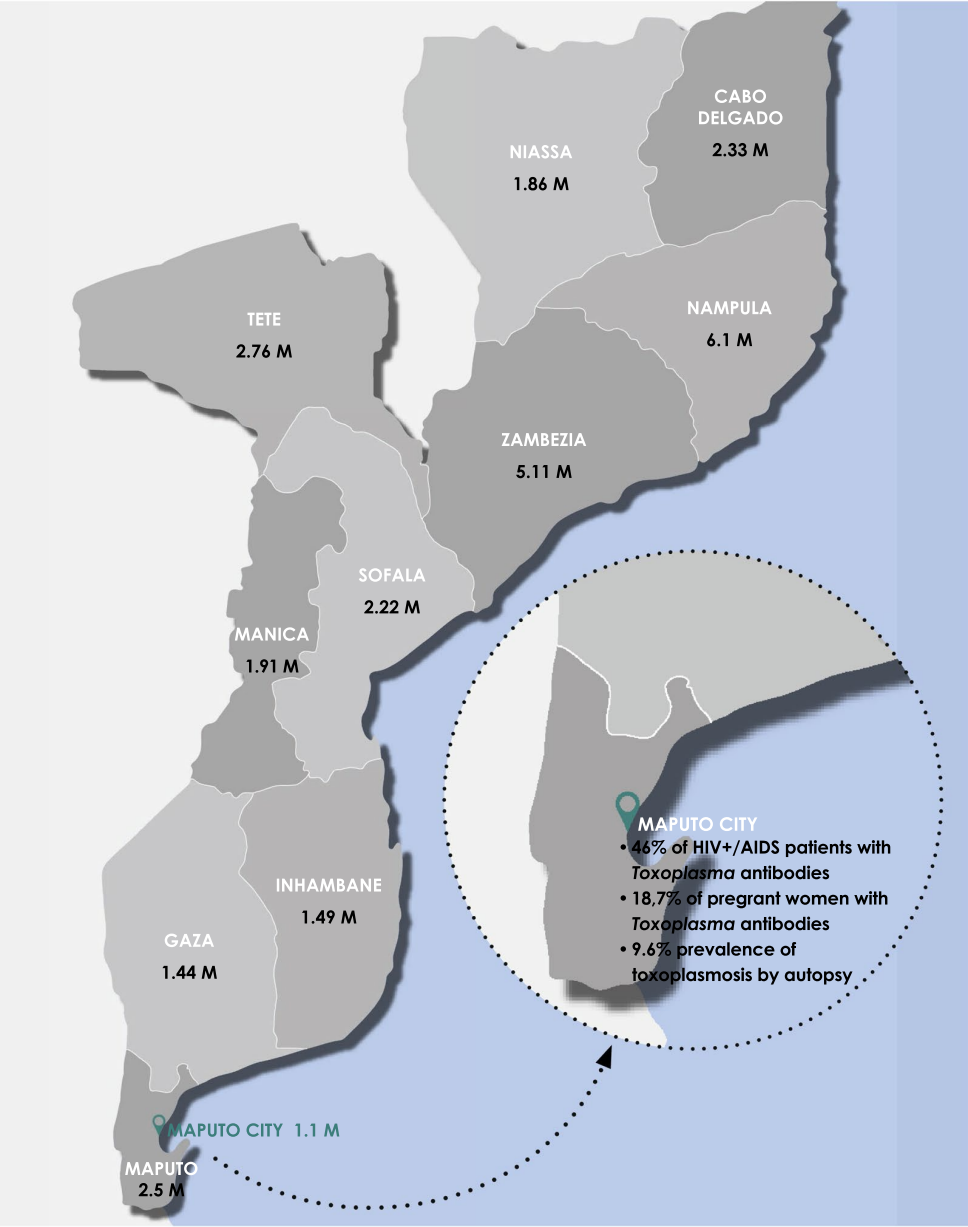
*Toxoplasma* studies reported in Mozambique. Map indicating the population of each Mozambique province following the 2017 census [85], and where the published studies were made. *Abbreviation*: M, millions

Although *T. gondii* is commonly associated with immunodeficiency disorders, a limited number of studies have been performed in Africa and, to the best of our knowledge, nobody has previously investigated the risk of reactivation of latent *T. gondii* infection in Mozambique HIV^+^ patients, for example. However, in the last decade a cross-sectional study in HIV^+^/AIDS patients performed in Maputo (Mozambique) found a 46% prevalence of anti-*Toxoplasma* IgG, pointing towards a latent *Toxoplasma* infection among almost half of HIV^+^/AIDS patients. This study also identified the regular consumption of cattle meat, breeding cats and dogs, and regular contact with the soil as risk factors to acquire *Toxoplasma* infection [11]. Another study aiming to assess clinic-pathological discrepancies in the diagnosis of causes of death in HIV infected adults from Mozambique found that none of the 8/73 (9.6%) cases of toxoplasmosis confirmed in the autopsy were clinically suspected, indicating 100% of major clinical discrepancies [105] (Fig. 1).

Congenital toxoplasmosis can cause miscarriage, blindness, deafness, hydrocephalus, and brain damage. Newborns may appear healthy at birth, but later in life can suffer from eye diseases, or have cognitive difficulties. Moreover, screening of *Toxoplasma* infection during pregnancy and specific treatment resulted in a decrease of *T. gondii* transmission from mother to child and a consequent reduction of clinical sequelae in infants [43]. There are no published data on the burden of congenital toxoplasmosis in Mozambique, nor its relation with hydrocephalus, seizures and mental retardation, which are disorders prevalent in Mozambique as already referred. A previous study performed by Sitoe et al. [106] in 150 pregnant women seeking the first trimester prenatal care in Maputo Central Hospital (Maputo, Mozambique) reported an overall prevalence of 18.7% of *Toxoplasma* IgG antibodies with the occurrence of 31.3% in HIV^+^ pregnant women and 10.9% in immunocompetent pregnant women (Fig. 1). Taking into account the dimension of this study, the findings indicate the less than 25% of the pregnant woman had previous contact with *T. gondii* and that part of these women were *Toxoplasma*/HIV co-infected. In the same study, only one woman presenting significant levels of anti-*Toxoplasma* IgM was found [106], pointing towards a lower occurrence of active *T. gondii* infection during the first trimester of pregnancy in Maputo. The incidence of neonatal hydrocephalus in Mozambique was estimated to be 3–5 cases per 1000 live births [102], which leads to severe disability as is a significant cause of morbidity and mortality. However, the proportion of children that acquired *T. gondii* infection during pregnancy and developed congenital toxoplasmosis is not known. Similarly, there are no studies available on the importance of *T. gondii* infection in ocular disease, mental retardation, schizophrenia and epilepsy either in children or in adults living in Mozambique.

Moreover, in Mozambique, there are no available data on the clinical expression and severity of human toxoplasmosis in the diverse segments of the population and across the different regions, identification of *T*. *gondii* strains that circulate in the country, and on the dynamics of parasite transmission.

## Conclusions

*Toxoplasma gondii* is a globally widespread parasite that invades and chronically persists in the central nervous system of infected individuals. The complexity of this infection is a consequence of the intimate relationship established between the parasite and the host immunity, which can lead to persistent infections of limited pathogenesis. Further advances in the understanding of *Toxoplasma* infection have found a link between this parasite, mental and mood disorders, and suicidal behavior. However, this parasitic infection should not be evaluated from a restricted anthroponotic perspective since wild and domestic animals are involved in parasite genetic recombination and transmission. Moreover, molecular studies contributed to identifying different parasite strains associated with virulence levels and implicated in the specific clinical characteristics of the disease. Thus, it is of utmost importance to estimate the incidence of *T. gondii* infection and toxoplasmosis among the Mozambique population. The few available studies reflect the reality of Maputo, the Mozambique capital, and comprehensive information for the entire country is missing. Furthermore, understanding the extension of *T. gondii* infection and the risk factors associated is a pre-requisite for the development of effective control measures. Then, it is urgent to develop and implement research programs directed to the gaps of knowledge, which include up-to-date information on *T. gondii* geographical distribution, the incidence and risk factors of immunocompetent, congenital, and perinatal infection, the occurrence of ocular toxoplasmosis, the association of *Toxoplasma* infection with mental and neurodegenerative disorders, as well as the identification of *T. gondii* genotypes circulating in Mozambique and its relation to morbidity and mortality. The research priorities identified for Mozambique can be an excellent starting point to inspire neighboring eastern African countries to foster research, in particular on this parasitic infection, and thus increasing knowledge in scientific and clinical areas, promoting the reduction of social impacts related to disability and consequent labor hours lost and advance the social-economic conditions.

## Data Availability

Not applicable.

## Abbreviations

AIDS: acquired immunodeficiency syndrome
DNA: deoxyribonucleic acid
HIV: human immunodeficiency virus
PCR-RFLP: polymerase chain reaction-restriction fragment length polymorphism
IgG: immunoglobulin G
IgM: immunoglobulin M
PCR: polymerase chain reaction
cART: combination antiretroviral therapy
DALYs: Disability-adjusted life years
